# Inhibition of Insulin Secretion Induces Golgi Morphological Changes

**DOI:** 10.14789/jmj.JMJ22-0040-OA

**Published:** 2023-02-22

**Authors:** TATSUYA IWAMOTO, SHIGEOMI SHIMIZU, HAJIME TAJIMA-SAKURAI, HIROFUMI YAMAGUCHI, YUYA NISHIDA, SATOKO ARAKAWA, HIROTAKA WATADA

**Affiliations:** 1 Department of Metabolism & Endocrinology, Juntendo University Graduate School of Medicine, Tokyo, Japan; 1 Department of Metabolism & Endocrinology, Juntendo University Graduate School of Medicine, Tokyo, Japan; 2 Department of Pathological Cell Biology, Medical Research Institute, Tokyo Medical and Dental University (TMDU), Tokyo, Japan; 2 Department of Pathological Cell Biology, Medical Research Institute, Tokyo Medical and Dental University (TMDU), Tokyo, Japan

**Keywords:** autophagy, pancreatic β cells, ER, Golgi, diazoxide

## Abstract

**Objectives:**

The role of autophagy in pancreatic β cells has been reported, but the relationship between autophagy and insulin metabolism is complex and is not fully understood yet.

**Design:**

We here analyze the relationship between autophagy and insulin metabolism from a morphological aspect.

**Methods:**

We observe the morphological changes of β cell-specific Atg7-deficient mice and Atg5-deficient MIN6 cells with electron microscopy.

**Results:**

We find that Atg7-deficient β cells exhibit a marked expansion of the endoplasmic reticulum (ER). We also find that the inhibitory state of insulin secretion causes morphological changes in the Golgi, including ministacking and swelling. The same morphological alterations are observed when insulin secretion is suppressed in Atg5-deficient MIN6 cells.

**Conclusions:**

The defect of autophagy induces ER expansion, and inhibition of insulin secretion induces Golgi swelling, probably via ER stress and Golgi stress, respectively.

## Introduction

Pancreatic β cells are specialized for the synthesis and secretion of insulin. Because insulin is a hormone that regulates glucose utilization, pancreatic β cells play an essential role in the body by efficiently mobilizing intracellular organelles. For example, mitochondria promote glucose-responsive insulin secretion via the ATP synthesis. Therefore, when mitochondria become dysfunction, insulin secretion is substantially reduced. In the rough endoplasmic reticulum (ER), preproinsulin is biosynthesized^1, 2)^. The pre-sequence corresponds to a signal peptide, which is cleaved after translocation into the ER to form proinsulin^3)^. After transport to the Golgi apparatus, proinsulin is included into insulin secretory granules and transported out of the cell. During this process, proinsulin is cleaved to become mature insulin in the secretory granules^4)^. In the β cell, the ER is continually subjected to high levels of ER stress in order to synthesize large amounts of insulin, and hence, further elevated stress enhances the ER stress response and induce cell death^5)^. Thus, various organelles function has been analyzed, whereas their morphological observations have not yet been fully analyzed.

Autophagy is a cellular function in which intracellular components are enclosed by a double membrane and are digested by fusing with lysosomes^6)^. Autophagic structures are considered as a type of organelle because they are consisted by bio-membranes and perform specific cellular functions. However, unlike other organelles, the number of autophagic structure fluctuates depending on the environment in which the cell is placed. In a steady state, a small amount of autophagic structures degrades small amount of proteins for maintaining cellular homeostasis. On the other hand, under stressful conditions, a large amount of autophagic structures are generated to degrade vast amount of proteins and even organelles^7)^. Many papers have reported the role of autophagy in pancreatic β cells. For example, induction of autophagy has been reported in pancreatic β cells of mice challenged with a high-fat diet and of type 2 diabetes model mice. In mice lacking Atg7, an essential molecule for autophagy, in pancreatic β cells, glucose-responsive insulin secretion becomes abnormal, and thereby, the mice showed hyperglycemia after 6 weeks of age^8)^. On the other hand, it has been reported that insulin granules are not a substrate for autophagy^9, 10)^. Therefore, the relationship between autophagy and insulin metabolism is complex and is not fully understood yet. In order to obtain data to solve this problem, we here analyzed the morphology of organelles in autophagy-deficient pancreatic β cells.

## Materials and Methods

### Mammalian cell culture

MIN6 cells were kindly provided by Prof. J. Miyazaki (Osaka University). Atg5 KO MIN6 cells were generated by the CRISPR/Cas9 system^10)^ (Cong et al., 2013). Briefly, a 20-bp mouse Atg5-targeting sequence (GAGAGTCAGCTATTTGACGT) was synthesized (Eurofins) and introduced into px330 (Addgene). MIN6 cells were co-transfected with the plasmid and pcDNA3.1 (Invitrogen), which contains the neomycin gene, and G418 selection (800 µg/ml) was initiated 24 hr later. After 48 hr selection, the MIN6 cells were re-seeded to allow single colony formation. The knockout of Atg5 was confirmed by anti-Atg5 immunoblot. Cells were grown in modified Dulbecco’s modified Eagle’s medium (DMEM high glucose, Nacalai) supplemented with 0.25 mM 2-mercaptoethanol, 55 U/mL penicillin, 55 g/mL streptomycin, and 11% (v/v) fetal bovine serum in a humidified 5% CO2 incubator at 37°C. For the analysis of glucose depletion, cells were stimulated in low glucose (135 mM NaCl, 3.6 mM KCl, 1.5 mM CaCl2, 0.5 mM NaH2PO4, 0.5 mM MgCl2, 2 mM NaHCO3, 10 mM HEPES, 0.1% BSA, and 2.5 mM glucose, pH 7.4) or control KRB buffer (135 mM NaCl, 3.6 mM KCl, 1.5 mM CaCl2, 0.5 mM NaH2PO4, 0.5 mM MgCl2, 2 mM NaHCO3, 10 mM HEPES, 0.1% BSA, and 25 mM glucose, pH7.4) after PBS wash.

### Mice

Atg7^flox/flox^: Rip-cre mice were generated by the crossbreeding of Atg7^flox/flox^ mice with Rip-cre mice^10, 11)^. To suppress insulin secretion, 50 mg/kg Diazoxide (SIGMA) was added by peritoneal administration for an hour before perfusion-fixation. Mice were bred in a 12 hr light /12 hr dark cycle at approximately 23°C and 40% relative humidity at the Laboratory for Recombinant Animals of Tokyo Medical and Dental University, Tokyo, Japan. This animal facility is operated according to the NIH guidelines. The Tokyo Medical and Dental University Ethics Committee for Animal Experiments approved all experiments in this study, and all experiments were performed according to their regulations.

### Electron microscopy

Mammalian cells were fixed by a conventional method (1.5% paraformaldehyde and 3% glutaraldehyde in 0.1 M phosphate buffer, pH 7.4, followed by an aqueous solution of 1% Osmium Tetroxide). Fixed samples were embedded in Epon 812, and thin sections (70-80 nm) were then cut and stained with uranyl acetate and lead citrate for observation under a Jeol-1010 electron microscope (Jeol) at 80 kV^12, 13)^.

## Results

### Morphological characteristics in wild-type pancreatic β cells

**Figure 1:**
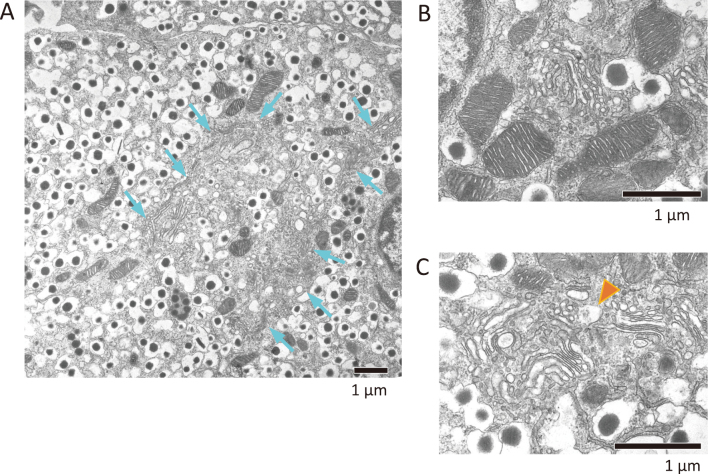
EM analysis of β cells from wild-type mice Islet is isolated from wild-type mice, and β cell was observed by EM. (A) The cytoplasm is filled with insulin granules. In the center of them, there is a Golgi apparatus (blue arrows). (B) Mitochondria are energized. (C) Ribbon shaped-Golgi apparatus is surrounded by insulin granules. Small-sized autophagic vacuole (orange arrowhead) is existed close to Golgi apparatus.

First, to analyze the morphological characteristics of pancreatic β cells, we isolated islets from wild-type mice and observed by electron microscopy. Unlike other sort of cells, a large amount of insulin granules were present inside the pancreatic β cells (Figure 1A). In addition, well-developed mitochondria, ER, and Golgi apparatus were observed (Figure 1B), all of which were considered to be actively functioning. Only a few autophagosome was observed (Figure 1C).

### Autophagy-deficient pancreatic β cells show inclusion body formation and endoplasmic reticulum expansion

**Figure 2:**
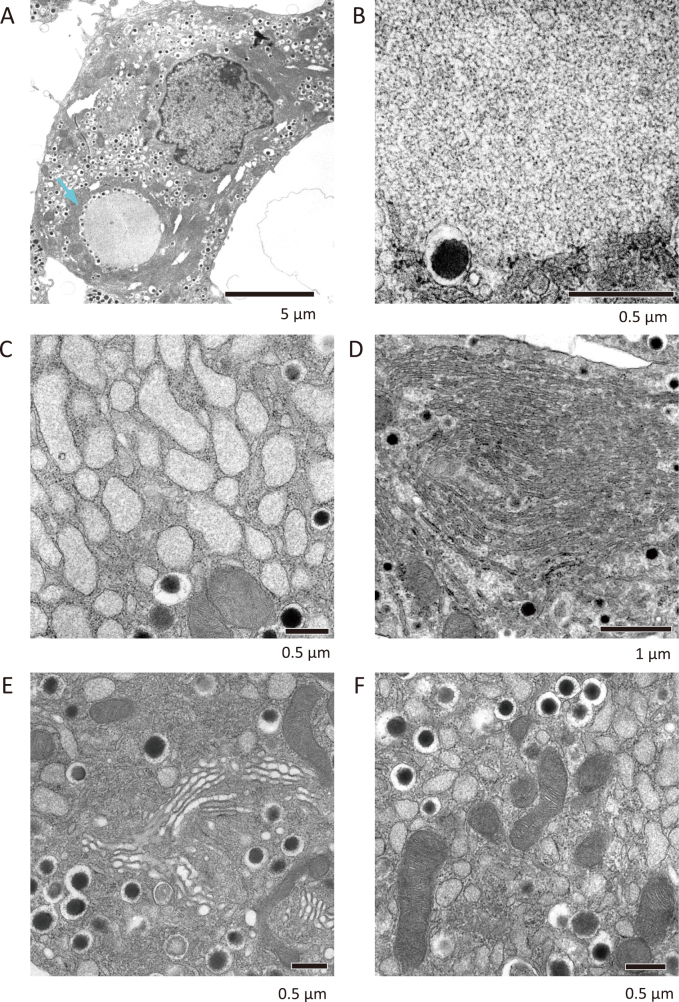
EM analysis of β cell from β cell-specific Atg7-deficient mice The pancreas from Atg7^flox/flox^: Rip-cre mouse was perfusion-fixed and β cell was observed by EM. (A) We observed representative inclusion body (blue arrow). Such structure was not observed in wild-type β cells. (B) Magnified image of inclusion body containing a dense meshwork of filamentous assemblies. (C, D) Abnormal morphology of the ER. (C) Rough ER with small number of ribosomes was remarkably swollen with fibrous structures. In (D), layered ER was also observed. (E, F) Normal morphology of the Golgi and mitochondria. Golgi apparatus (E) and mitochondria (F) were morphologically intact.

Next, we generated mice lacking Atg7 in pancreatic β cells by cross-breeding Atg7^flox/flox^ mice with Rip-cre mice^10, 11)^. These mice have been shown to have pancreatic β cells in which autophagy does not occur. We observed Atg7-deficient β cells by electron microscopy, and there was no significant difference in the amount or location of insulin granules from wild-type β cells (Figure 2A). On the other hand, the most significant change was the presence of inclusion bodies (Figure 2A), whose internal structure was a dense meshwork of filamentous assemblies (Figure 2B), in a large number of cells. Because p62 is known to accumulate in Atg7-deficient β cells and the electron microscopic findings are completely identical to the previously reported p62 granules^14)^, this inclusion body was considered to be a p62 granule.

Another significant morphological change was observed in the ER, which was observed to have a bulging lumen and a small amount of ribosomes attached to it (Figure 2C), as well as layered ER (Figure 2D). Such abnormal ER morphology is often observed in the cells with excess ER stress^15)^. The structures within the ER lumen are fibrous (Figure 2C), and judging by their shape, they were suspected to be unfolded polypeptides. In pancreatic β cells, at least, proinsulin or islet amyloid polypeptide (IAPP), a peptide consisting of 37 residues^16)^, were considered to be accumulated in the ER lumen. Furthermore, because IAPP, but not insulin, is degraded by autophagy^10, 17)^, IAPP could be one of the structures in the ER of Atg7-deficient β cells. No major abnormalities were observed in the Golgi apparatus or mitochondria (Figure 2E, F).

### Inhibition of insulin secretion causes Golgi morphology abnormalities

**Figure 3:**
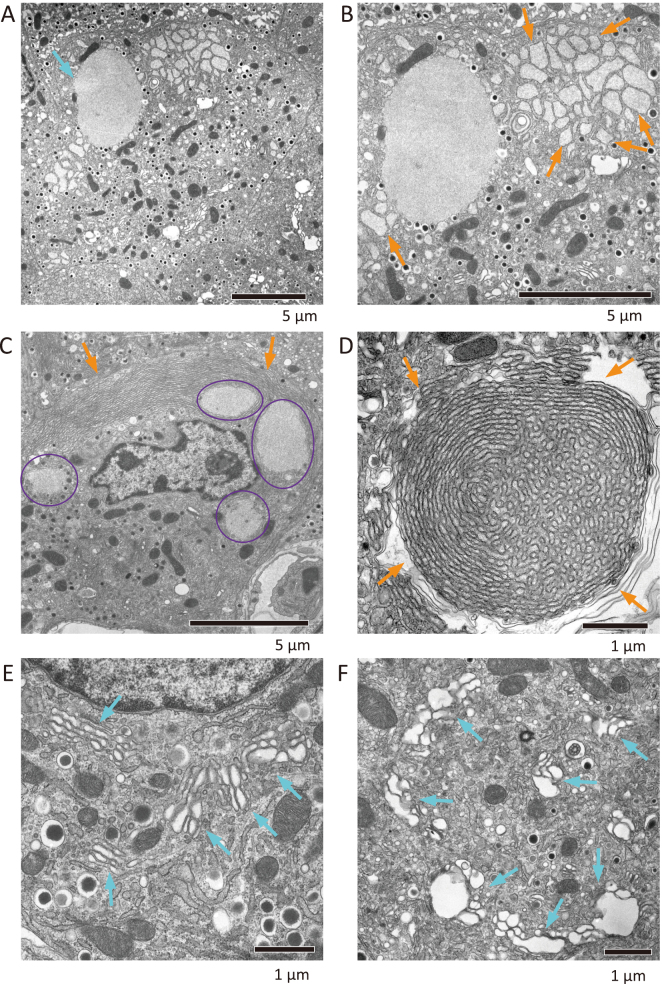
EM analysis of β cell from β cell-specific Atg7-deficient mice upon diazoxide treatment Diazoxide was treated intraperitoneally to Atg7^flox/flox^: Rip-cre mice for 1 hour. Then, the mice were perfusion-fixed and β cells were observed by EM. (A) Inclusion body was enlarged (blue arrow). (B) Magnified image of (A). Inclusion body contained a dense meshwork of filamentous assemblies, and rough ERs were remarkably swollen (orange arrows). (C) The four inclusion bodies (purple circles) and layered ER (orange arrows) were observed. In (D), layered ERs were assembled in a whirlpool (orange arrows). (E, F) Abnormal morphology of the Golgi apparatus. Golgi apparatus were separated into mini-stacks (blue arrows in E) and swollen (blue arrows in F).

Diazoxide, a KATP channel activator, is used as a therapeutic agent for insulinoma because of its pharmacological effect of inhibiting insulin secretion^18)^. When Atg7^flox/flox^: RIP-Cre mice were treated with Diazoxide to suppress insulin secretion, the number and size of inclusions increased, with a diameter of nearly 5 µm (Figure 3A, B). In some cases, four inclusions were found in a single cell (Figure 3C). The abnormalities of the ER became more pronounced, with the appearance of ERs with progressive lumenal swelling (Figure 3B) and spiral-like stratified ERs (Figure 3D), suggesting that ER stress had progressed. In addition, abnormalities in Golgi morphology were newly observed; mini stacks of the Golgi and swelling of the cisterna (Figure 3E, F), which should be induced by the accumulation of insulin granules in the Golgi apparatus.

### MIN6 cells also show Golgi deformation under insulin secretion inhibition

**Figure 4:**
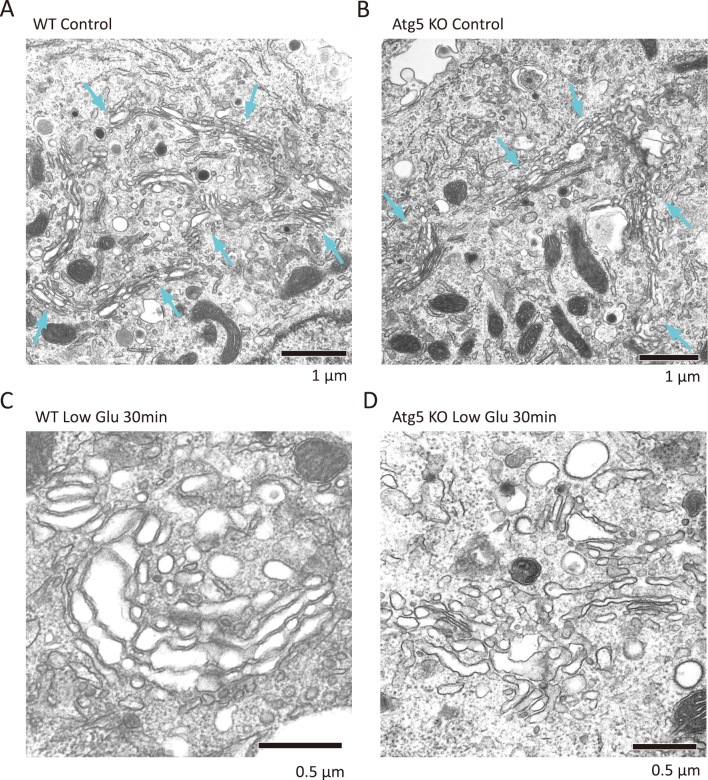
EM analysis of MIN6 cells (A, B) EM analysis revealed no difference between wild-type MIN6 cells (A) and Atg5-deficeint MIN6 cells (B). In both cells, Golgi apparatus (blue arrows) localized at the center of the cells. (C, D) Glucose deprivation generated mini-stacked Golgi and Golgi swelling both in wild-type MIN6 cells (C) and Atg5-deficeint MIN6 cells (D).

MIN6 cells are a glucose-responsive insulin-secreting cell line derived from mouse pancreatic β cells^19)^. Absence of Atg5, a molecule required for autophagy execution as well as Atg7, in this cell line did not show any abnormal structures, including inclusion body formation and a bulging ER lumen, unlike Atg7-deficient pancreatic β cells (Figure 4A, B). This difference may be due to the difference between in vivo β cells and lineage β cells. The Golgi apparatus was also normal in morphology under normal culture condition (Figure 4A, B). However, when the glucose concentration was lowered and insulin secretion was suppressed^10)^, as similar situation with Diazoxide-treatment, Golgi was mini-stacked and swelling of the cisterna were evident both in wild-type MIN6 cells and Atg5-deficient MIN6 cells (Figure 4C, D). These results suggest that the alteration of Golgi morphology is induced by suppression of insulin secretion in MIN6 cells like pancreatic β cells.

## Discussion

In this study, we analyzed the influence of autophagy in pancreatic β cells from a morphological aspect. We found that the analysis of Atg7-deficient β cells revealed a pronounced ER dilatation, which is generally induced by the abnormality of protein turnover. In the case of β cells, insulin and IAPP, which are specifically synthesized in β cells, are thought to be involved in this morphological alteration. However, insulin degradation involves proteolytic systems distinct from autophagy, called stress- induced nascent granule degradation (SINGD)^9)^ and Golgi membrane-associated degradation (GOMED)^10, 12, 20, 21)^. SINGD is a cellular function in which newly synthesized insulin granules are degraded by direct fusion with lysosomes, while GOMED is a function in which insulin granules are wrapped in the Golgi membrane followed by the degradation after fusion with lysosomes. Unlike SINGD and GOMED, autophagy has been reported to play little role with respect to insulin degradation, suggesting that insulin might not be involved in the morphological alternation of the ER. On the other hand, with regard to IAPP, it was reported that Atg7 deficiency in human IAPP-expressing mice increased IAPP amyloid and caused glucose intolerance^22)^, indicating that IAPP is a substrate for autophagy degradation. Therefore, IAPP might be involved in the ER alteration induced by autophagy deficiency.

In pancreatic β cells, IAPP is degraded by autophagy, while insulin granules are not. This finding demonstrated the substrate specificity of autophagy, and there are many other similar examples. For example, in the final differentiation of erythrocytes, ribosomes, but not mitochondria, are degraded by autophagy^21, 23)^. Although the precise reason of different sensitivity against autophagy between IAPP and insulin is unidentified, this might be owing to the recognition by autophagic adaptor proteins.

In this study, we find that inhibition of insulin secretion causes morphological changes in the Golgi apparatus. When the transport of molecules, including insulin, to the extracellular and plasma membrane via the Golgi is blocked, Golgi is usually deformed to become ministack-Golgi and swelling. The cause of Golgi deformation is thought to be the simultaneous morphological changes required to execute GOMED to degrade the retained molecules, in addition to the changes that occur passively due to molecular retention in the Golgi^10, 20)^. Specifically, Golgi swelling appears to be a passive response owing to substance retention, while mini-stacking is a process necessary for GOMED execution.

## Funding

This study was supported in part by Grant-in-Aid for Scientific Research (A) (17H01533, 20H00467), Grant-in-Aid for Scientific Research on Innovative Areas (17H06414, 22H04899, 22H04639) from the MEXT of Japan. This study was also supported by AMED under Grant Number JP22wm0525028 and JP22gm1410012, and by the Joint Usage/Research Program of Medical Research Institute, Tokyo Medical and Dental University.

## Author contributions

S S, H S and S A designed the experiments, developed the workflow, analyzed the data. T I, H S, H Y, H Y and S K performed the experiments. S S, Y N and H W wrote the manuscript; Y N and H W participated in study conceptualization and helped improve the manuscript. All authors read and approved the final manuscript.

## Conflicts of interest statement

The authors declare that there are no conflicts of interest.

## References

[1] Chan SJ, Keim P, Steiner DF: Cell-free synthesis of rat preproinsulins: characterization and partial amino acid sequence determination. Proc Natl Acad Sci USA. 1976; 73: 1964-1968. 778852 10.1073/pnas.73.6.1964 PMC430428

[2] Bell GI, Pictet RL, Rutter WJ, Cordell B, Tischer E, Goodman HM: Sequence of insulin gene. Nature. 1980; 284: 26-32. 6243748 10.1038/284026a0

[3] Patzelt C, Labrecque A., Duguid J, et al: Detection and kinetic behavior of preproinsulin in pancreatic islets. Proc Natl Acad Sci USA. 1978; 75: 1260-1264. 206890 10.1073/pnas.75.3.1260 PMC411450

[4] Smeekens SP, Montag AG, Thomas G, et al: Proinsulin processing by the subtilisin-related proprotein convertases furin, PC2 and PC3. Proc Natl Acad Sci USA. 1992; 89: 8822-8826. 1528899 10.1073/pnas.89.18.8822 PMC50013

[5] Yong J, Johnson JD, Arvan P, Han J, Kaufman RJ: Therapeutic opportunities for pancreatic β cell ER stress in diabetes mellitus. Nat Rev Endocrinol. 2021; 17: 455-467. 34163039 10.1038/s41574-021-00510-4 PMC8765009

[6] Mizushima N, Komatsu M: Autophagy: renovation of cells and tissues. Cell. 2011; 147: 728-741. 22078875 10.1016/j.cell.2011.10.026

[7] Liu K, Sutter BM, and Tu BP: Autophagy sustains glutamate and aspartate synthesis in Saccharomyces cerevisiae during nitrogen starvation. Nat Commun. 2021; 12: 57. 33397945 10.1038/s41467-020-20253-6 PMC7782722

[8] Ebato C, Uchida T, Arakawa M, et al: Autophagy is important in islet homeostasis and compensatory increase of beta cell mass in response to high-fat diet. Cell Metab. 2008; 8: 325-332. 18840363 10.1016/j.cmet.2008.08.009

[9] Goginashvili A, Zhang Z, Erbs E, et al: Insulin granules. Insulin secretory granules control autophagy in pancreatic β cells. Science. 2015; 347: 878-882. 25700520 10.1126/science.aaa2628

[10] Yamaguchi H, Arakawa S, Kanaseki T, et al: Golgi membrane-associated degradation pathway in yeast and mammals. EMBO J. 2016; 35: 1991-2007. 27511903 10.15252/embj.201593191 PMC5282831

[11] Lee JY, Ristow M, Lin X, White MF, Magnuson MA, Hennighausen L: RIP-Cre revisited, evidence for impairments of pancreatic beta-cell function. J Biol Chem. 2006; 281: 2649-2653. 16326700 10.1074/jbc.M512373200

[12] Yamaguchi H, Honda S, Torii S, et al: Wipi3 is essential for alternative autophagy and its loss causes neurodegeneration. Nat Commun. 2020; 11: 5311. 10.1038/s41467-020-18892-w PMC7576787 33082312

[13] Arakawa S, Tsujioka M, Yoshida T, et al: Role of Atg5-dependent cell death in the embryonic development of Bax/Bak double-knockout mice. Cell Death Differ. 2017; 9: 1598-1608. 10.1038/cdd.2017.84 PMC5563990 28574506

[14] Fukae T, Miyatsuka T, Himuro M, et al: Genetic ablation of p62/SQSTM1 demonstrates little effect on pancreatic β cell function under autophagy deficiency. Biochem Biophys Res Commun. 2022; 612: 99-104. 35512463 10.1016/j.bbrc.2022.04.092

[15] Wang Y, Osakue D, Yang E, et al: Endoplasmic Reticulum Stress Response of Trabecular Meshwork Stem Cells and Trabecular Meshwork Cells and Protective Effects of Activated PERK Pathway. Invest Ophthalmol Vis Sci. 2019; 60: 265-273. 30654386 10.1167/iovs.18-25477 PMC6340162

[16] Westermark P, Andersson A, Westermark GT: Islet amyloid polypeptide, islet amyloid, and diabetes mellitus. Physiol Rev. 2011; 91: 795-826. 21742788 10.1152/physrev.00042.2009

[17] Kim J, Park K, Kim MJ, et al: An autophagy enhancer ameliorates diabetes of human IAPP-transgenic mice through clearance of amyloidogenic oligomer. Nat Commun. 2021; 12: 183. 33420039 10.1038/s41467-020-20454-z PMC7794419

[18] Okabayashi T, Shima Y, Sumiyoshi T, et al: Diagnosis and management of insulinoma. World J Gastroenterol. 2013; 19: 829-837. 23430217 10.3748/wjg.v19.i6.829 PMC3574879

[19] Miyazaki J, Araki K, Yamato E, et al: Establishment of a pancreatic beta cell line that retains glucose-inducible insulin secretion: special reference to expression of glucose transporter isoforms. Endocrinology. 1990; 127: 126-132. 2163307 10.1210/endo-127-1-126

[20] Nishida Y, Arakawa S, Fujitani, K, et al: Discovery of Atg5/Atg7-independent alternative macroautophagy. Nature. 2009; 461: 654-658. 19794493 10.1038/nature08455

[21] Honda S, Arakawa S, Nishida Y, Yamaguchi H, Ishii E, Shimizu S: Ulk1-mediated Atg5-independent macroautophagy mediates elimination of mitochondria from embryonic reticulocytes. Nat Commun. 2014; 5: 4004. 24895007 10.1038/ncomms5004

[22] Kim J, Cheon H, Jeong, YT, et al: Amyloidogenic peptide oligomer accumulation in autophagy-deficient β cells induces diabetes. J Clin Invest, 2014; 124: 3311-3324. 25036705 10.1172/JCI69625 PMC4109549

[23] Mortensen M, Ferguson DJ, Edelmann M, et al: Loss of autophagy in erythroid cells leads to defective removal of mitochondria and severe anemia in vivo. Proc Natl Acad Sci USA. 2010; 107: 832-837. 20080761 10.1073/pnas.0913170107 PMC2818953

